# F90. SOCIAL INFERENCE AND BELIEFS DIFFER IN INDIVIDUALS WITH SUBCLINICAL PERSECUTORY DELUSIONAL TENDENCIES

**DOI:** 10.1093/schbul/sby017.621

**Published:** 2018-04-01

**Authors:** Katharina Wellstein, Andreea Diaconescu, Martin Bischof, Annia Rüesch Ranganadan, Johannes Ullrich, Klaas Enno Stephan

**Affiliations:** 1 ETH Zurich; 2 University of Zurich, ETH Zurich; 3 Psychiatric University Hospital Zurich; 4 University of Zurich

## Abstract

**Background:**

It has long been suspected that abnormalities in social inference (e.g., about the intentions of others) play a key role for persecutory delusions. In this study, we examined the association between subclinical persecutory delusions (PD) and social inference, testing the prediction that proneness to PD is related to altered social inference and beliefs.

**Methods:**

We included 148 participants who scored on opposite ends of Freeman’s Paranoia Checklist (PCL). High scorers and low scorers were thus assigned to two respective participant groups, which were matched according to age, education in years, and gender. Participants performed a probabilistic advice-taking task with a dynamically changing social context (volatility) under one of two experimental frames. Our design was thus 2x2 factorial (high vs. low delusional tendencies, dispositional vs. situational frame). In the task, participants had to integrate two types of cues simultaneously in order to make informed predictions, namely a social cue (advice provided by an adviser) and a non-social cue (probabilities given via pie-chart). In addition, the experimental frames differentially emphasized possible reasons behind unhelpful advice and either highlighted (i) the adviser’s possible intentions (dispositional frame) or (ii) the rules of the game (situational frame). Task structure was identical across frames. When integrating the framing information, participants were expected to take advice into account more in the situational frame than in the dispositional frame, since the latter induces some mistrust due to highlighting the adviser’s intentions.

**Results:**

The behavioral data showed significant group-by-frame interactions (F=5.7381, p<0.05), indicating that in the situational frame high PCL scorers took advice less into account than low scorers. This reduced adaptation to the frame was particularly visible after the experience of volatility. Additionally, high PCL scorers believed significantly more frequently that incorrect advice was delivered intentionally (F=16.369, p<0.001) and that such malevolent behavior was directed towards them personally (p<0.05). High scorers also reported attributing unhelpful advice more to the adviser (F=8.047, p<0.01) instead of the rules of the game, compared to low scorers. The high scorers in the PCL reported higher negative, positive, and depressive symptoms on the CAPE compared to low scorers (p<0.001) but did not differ regarding cognitive performance in the Brief Neurocognitive Assessment (BNA).

**Discussion:**

Overall, our results suggest that social inference in individuals with subclinical PD tendencies is less sensitive to differences in social context and shaped by negative beliefs about the intentions of others. These findings may help future attempts of identifying at risk mental state individuals and understanding maladaptive behavior in schizophrenia.

